# Importance of Publication Audit, fast track processing and categorization of manuscripts

**DOI:** 10.12669/pjms.332.12858

**Published:** 2017

**Authors:** Shaukat Ali Jawaid

Publication audit is a very useful tool to evaluate progress of a journal and plan for the future.^1^ We have been doing this exercise for the last quite a few years. During the Year 2016 we received 1703 manuscripts of which three hundred twenty one were published, 1386 were not accepted for further processing due to various reasons, two were rejected because of plagiarism while twelve were withdrawn by the authors. Majority of the manuscripts from Pakistan as expected were submitted from Karachi (116) followed by Lahore (87) and Islamabad (26). Table-I, Table-II, Table-III. It must be mentioned here that we only screen those manuscripts for plagiarism which are accepted for further processing after initial internal review but before they are sent for external review. Some of these authors are advised to rewrite their manuscripts to reduce the similarity index score to come within acceptable limit of less than 20% before they are accepted for further processing. Original articles were the largest category of manuscripts published during 2016(Table-IV). Details regarding the name of the countries from which we received manuscripts for publication during the Year 2016 are given in Table-V.

**Table-I:** Country wise submissions during 2016.

*Country*	*Total*
Australia	1
Bangladesh	2
China	572
Serbia and Montenegro	2
Cyprus	1
Germany	1
Algeria	3
Egypt	8
France	3
United Kingdom	4
Heard Island and McDonald Islands	1
Indonesia	7
Ireland	1
India	15
Iraq	11
Iran	112
Jordan	3
Republic of Korea	20
Kazakhstan	4
Lao People’s Democratic Republic	1
Sri Lanka	1
Macedonia	2
Malaysia	14
Nigeria	3
Nepal	5
New Zealand	1
Philippines	1
**Pakistan**	347
Poland	2
Saint Pierre and Miquelon	1
Palestinian	3
Qatar	1
Romania	9
Saudi arabia	80
Thailand	1
Turkey	450
Taiwan	3
United States of America	3
South Africa	1
Japan	1
Ethiopia	1
Azerbaijan	1

Grand Total	1703

**Table-II:** City wise submissions from Pakistan during 2016.

*City*	*Total*
Abbottabad	2
Bahawalpur	2
Bannu	1
Charsada	1
Dera Ismail Khan	2
Faisalabad	15
Gilgit	2
Gujrat	3
Havelian	1
Hyderabad	7
Islamabad	26
Jamshoro	1
Karachi	116
Khairpur	1
Lahore	87
Malakand	1
Mansehra	2
Mardan	4
Multan	14
Muzaffarabad	1
Peshawar	27
Quetta	2
Rahim Yar Khan	3
Rawalpindi	19
Sahiwal	1
Sargodha	5
Toba Tek Singh	1

Grand Total	347

**Table-III:** PJMS manuscripts statistics of 2016 at a Glance.

*Total Articles Published:*	*321*
Total Articles Rejected:	1386
Rejected because of plagiarism:	2
Articles withdrawn by authors:	12
Under Process:	18


Total Articles Received:	1703

**Table-IV:** Category Wise Manuscript Published in 2016.

*Category*	*Jan-Feb 2016*	*Mar-Apr 2016*	*May-Jun 2016*	*Jul-Aug 2016*	*Sep-Oct 2016*	*Nov-Dec 2016*	*Total*
Original Articles	50	48	48	45	45	47	283
Case Reports	2	2	4	3	2	2	15
Review Article	3	3	1	2	1	1	11
Editorial	1	1	0	1	1	0	4
Cont. Med Educ.	0	1	0	0	1	0	2
Short Comm.	0	0	1	0	1	0	2
Clinical Case Series	0	0	0	0	0	1	1
Conference Proceedings	0	0	0	1	0	0	1
Special Comm.	0	0	1	0	0	0	1
Systematic Review	0	0	0	0	0	1	1

Grand Total	56	55	55	52	51	52	321

**Table-V:** Country Wise Manuscript Published in 2016.

*Country*	*Jan-Feb 2016*	*Mar-Apr 2016*	*May-Jun 2016*	*Jul-Aug 2016*	*Sep-Oct 2016*	*Nov-Dec 2016*	*Total*
Pakistan	20	27	20	25	20	23	135
China	12	10	11	8	14	11	66
Turkey	11	8	14	12	9	9	63
Saudi Arabia	4	3	3	3	3	7	23
Iran	6	1	1	1	1	1	11
Korea	1	0	4	0	2	0	7
Malaysia	1	2	0	2	1	0	6
Australia	0	0	1	0	0	0	1
Bangladesh	0	0	1	0	0	0	1
United Kingdom	1	0	0	0	0	0	1
India	0	0	0	1	0	0	1
Iraq	0	1	0	0	0	0	1
Nigeria	0	1	0	0	0	0	1
Philippines	0	0	0	0	0	1	1
Romania	0	1	0	0	0	0	1
South Africa	0	0	0	0	1	0	1
Algeria	0	1	0	0	0	0	1

Total	56	55	55	52	51	52	321

**Fig.1:**
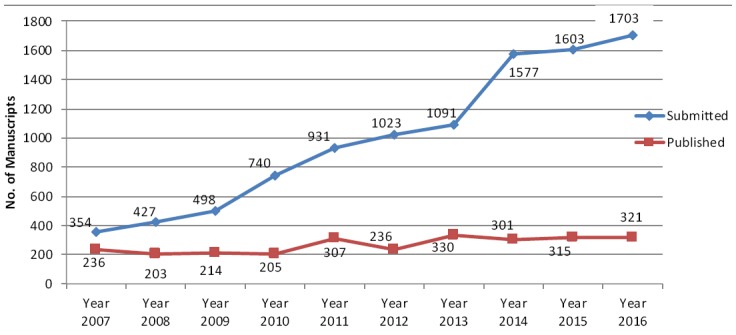
Number of manuscripts submitted and published in Pakistan Journal of Medical Sciences 2007-2016.

Last ten year’s analysis shows that our acceptance rate has constantly reduced from 50% in 2006 to just about under 20% in 2016. In fact it has remained within 20% over the last many years simply because we have an ever increasing number of submissions thus providing us an opportunity to be selective and accept only high quality manuscripts relevant to our readership in South East Asia and Asia Pacific region on which we have been concentrating. Fig.1. This also does not mean that those papers which are not accepted for further processing are all not of good quality but it is because of our financial and human resource limitations. We at present cannot process more than 300-350 manuscripts in a year. We publish six issues in a year and try to accommodate about fifty manuscripts in each issue. It is because of lot of pressure on limited space available that we request our authors to abide by the word limit set for each category of manuscript. We accept up to three thousand words in original article, fifteen hundred words for case reports and about four thousand words in Reviews. We always request the authors not to submit us the KAP Studies, Survey reports, animal studies since they have a very low priority with us. We are also much selective in accepting Meta-Analysis and Systemic Reviews, special communications. Since processing fee for Pakistan authors is non-refundable, if they have any doubt, they can check with us by sending an e mail with abstract to find out if their manuscript is likely to be accepted for further processing and publication or not. This will not only save their own precious time but our as well.

In order to help the authors, we changed our frequency from Quarterly publication to Bi-monthly publication from April-2013. We are visible on the PubMed/Midline through PubMed Central and we are indexed by SCIE ISI Thompson Reuter Web of Sciences. We had the privilege of having the highest Impact Factor among the three Pakistani medical journals as per Journal Citation Report issued by ISI Thompson Reuters last year. We continue our efforts to further improve the standard of the journal.

Original articles are the largest category of manuscripts published but in the absence of any universally accepted criteria regarding categorization of manuscripts, there exists a great disparity in manuscripts published by various journals. It is mostly the Editor’s discretion and there is likelihood of personal bias. Some of these papers that are published as original article can fit into Clinical Case Series or short communications but since the rules framed by regulatory agencies for selection and promotion of faculty members give credit to only original manuscripts, they are published as original as the editors do not wish to create problems for the authors. In the past we had suggested that let the PAME or a few selected leading biomedical journals of Pakistan come up with some guidelines on categorization and start implementing them. These guidelines will not be mandatory but advisory in nature and the Editors should be free to formulate their editorial policy for their respective journals.^2^ However, it carries the risk that if the institutions do not give equal credit to original studies published under the heading of original articles and Clinical Case Series etc., the authors will suffer, that is why the authors are always keen to get their manuscripts published as original articles. This issue is constantly being discussed in Pakistan by other Editor colleagues as well. This race for publishing original articles was neither a good practice nor in line with the recognized international standards.^3^-^4^ Randomized Clinical Trials (RCTs) is considered as the best and good quality of research but it is unfortunate that till today we do not have our own Trial Registry though our neighboring countries India and Iran has established their Trial Registries. This fact has also been earlier highlighted by Akhtar Sherin in an Editorial.^5^ Pakistan Medical Research Council sometime ago had taken the initiative to set up such a Trial Registry but no worthwhile progress has been made in this direction so far.
